# *Womandla Health*: development and rationale of a behavioral intervention to support HIV treatment adherence among postpartum women in South Africa

**DOI:** 10.1186/s12905-023-02817-y

**Published:** 2023-12-06

**Authors:** Jennifer A. Pellowski, Destry Jensen, Nokwazi Tsawe, Christopher Colvin, Susan Cu-Uvin, Don Operario, Mark Lurie, Abigail Harrison, Landon Myer, Lucia Knight

**Affiliations:** 1 School of Public Health, Brown University, Providence, USA; 2 School of Public Health, University of Cape Town, Cape Town, South Africa; 3 School of Medicine, University of Virginia, Charlottesville, USA; 4 The Warren Alpert Medical School of Brown University, Providence,, USA; 5 Rollins School of Public Health, Emory University, Atlanta, Georgia; 6 School of Public Health, University of the Western Cape, Cape Town, South Africa

**Keywords:** HIV, Pregnancy, Postpartum, Behavioral intervention

## Abstract

**Background:**

While Option B + has made great strides in eliminating vertical transmission of HIV and improving access to lifelong antiretroviral therapy (ART) for women, the postpartum period remains a risk period for disengagement from HIV care and non-adherence.

**Methods:**

Longitudinal qualitative data was collected from 30 women living with HIV in Cape Town, South Africa from pregnancy through 1 year postpartum to examine key barriers and facilitators to HIV treatment adherence across this transition. Participants were also asked about their preferences for behavioral intervention content, format, and scope. The intervention development process was guided by Fernandez et al.’s Intervention Mapping process and was informed by the qualitative data, the wider literature on ART adherence, and Transition Theory.

**Results:**

The *Womandla Health* Intervention is a multicomponent intervention consisting of four individual sessions with a lay health worker and four peer group sessions, which span late pregnancy and early postpartum. These sessions are guided by Transition Theory and utilize motivational interviewing techniques to empower women to ascertain their own individual barriers to HIV care and identify solutions and strategies to overcome these barriers.

**Conclusions:**

This intervention will be tested in a small scale RCT. If successful, findings will provide an innovative approach to HIV treatment by capitalizing on the transition into motherhood to bolster self-care behaviors, focusing on ART adherence and also women’s overall postpartum health and psychosocial needs.

## Introduction

Over 30% of women attending public antenatal clinics are HIV positive, compared to the national HIV prevalence rate among adults of 19% in South Africa [1, 2]. During pregnancy and postpartum, women living with HIV are eight times more likely to die than women who are HIV negative [3]. Prevention of mother-to-child transmission (PMTCT) efforts have reduced rates of vertical transmission to 2.7% in South Africa, [4] however, optimal postpartum adherence to HIV treatment, a key component of PMTCT, remains difficult [5–9]. Option B + policies, which provide antiretroviral therapy (ART) to all HIV positive pregnant women for life, regardless of CD4 T cell count, have been adopted in many countries as a way to simplify health services and increase access and uptake [9, 10]. There is major concern, however, about suboptimal engagement in care postpartum after mothers transfer from antenatal clinics to regular adult HIV care and the ability for women to adjust to lifelong treatment. In Cape Town, South Africa, 49% of women living with HIV have disengaged from care or missed an HIV care visit by six months postpartum [11] and a meta-analysis [5] of 51 global studies found that only 53% of the women had optimal adherence postpartum. Additionally, during the postpartum period, there is still ongoing risk for vertical transmission through breastfeedings, further underscoring the importance of high treatement adherence among this population [12]. To fully capitalize on the potential that Option B + has for simplifying HIV treatment, it is necessary to understand HIV treatment adherence during the 12 months following birth and how factors that influence adherence may change as HIV positive mothers transition from pregnancy to postpartum.

Pregnant and postpartum women are faced with a number of individual, contextual, and systems-level facilitators and barriers to HIV treatment adherence [13, 14]. Evidence shows lower education, [15] difficulties managing the practical demands of ART, [16] perceptions of being healthy, and the use of alcohol and drugs [17, 18] during pregnancy or postpartum are associated with suboptimal adherence. A major gap in evidence, however, is that previous studies rarely distinguish between pregnancy and postpartum, providing little information about the changes that may occur during this crucial transitional window period [14]. Additionally, balancing self-care with infant care, changes in familial roles, and finding out the infant’s HIV status are all associated with this transition and may influence adherence; however, discussion of these factors is minimal in the ART adherence literature [14]. Furthermore, interventions aimed at the general population are unlikely to meet the needs of postpartum women who are experiencing unique individual and contextual barriers and who therefore require tailored interventions [14]. Globally, ART adherence interventions tailored specifically for pregnant and postpartum women are severely lacking [19–21].

To fully realize the benefits of Option B + we aim (1) to understand how these dynamic factors influence HIV treatment and care across these transitions and (2) to develop an intervention that effectively addresses these moments of transition to bolster ART adherence for mothers living with HIV. This study details the formative qualitative work, intervention development, and rationale for a behavioral intervention for pregnant and postpartum women living with HIV in South Africa called *Womandla Health*.

## Theoretical framework

The formative qualitative work and intervention development draws on Transition Theory as an overarching theoretical framework. Transition Theory is a theory which originated in nursing that posits that individuals in transition may be particularly susceptible to risks that impact their health [22, 23]. Transition Theory allows us to characterize and describe the transitions that occur during this window period by clearly delineating the relevant personal and environmental characteristics that may facilitate or impede a successful transition, which could lead to sub-optimal ART adherence. With Transition Theory, we can conceptualize the pregnancy and postpartum period among women living with HIV as a time with multiple related health and developmental transitions. Women experience transition into motherhood (for a first child) or transitions in family roles (for a subsequent child). These women will also experience other transitions such as receiving the results of their infants’ HIV PCR test and shifting their own healthcare from antenatal clinics to HIV care clinics. Each of these transitions can present significant challenges for ART adherence. There are also properties of transition experiences that contribute to how well an individual is able to adhere to ART, including awareness about an impending transition – such as being aware of the need to transfer care to an HIV care clinic – and engagement in the processes inherent in the transition – such as seeking out information about infant care. Transition experiences influence outcomes, such as adherence to ART, via transition conditions. Transition conditions are personal and environmental factors that can facilitate or inhibit progress towards a successful transition. These conditions are: (a) personal meanings, including appraisals of the anticipated event and the effect it will have on an individual’s life, (b) cultural beliefs and attitudes, (c) socioeconomic status, (d) preparation for the transition, such as actively preparing for the infants’ arrival, (e) community factors including family and partner support and (f) societal factors such as marginalization of people living with HIV.

Transition Theory was used to guide the formative qualitative work in the Pregnancy, Adherence, Motherhood (PAM) Study [24, 25] and explicitly informed the design of data collection (longitudinal data collection spanning late pregnancy to 12 months postpartum) and the semi-structured interview content focusing on key transition experiences, conditions, and outcomes. The purpose of this study was to identify and characterize factors that influence pregnant and postpartum women’s adherence behaviors as well as perceptions and motivations for continued treatment and engagement in care and examine participants’ preferences for adherence intervention structure and delivery, in order to shape the design components of the bio-behavioral intervention.

## Methods of the PAM study

The PAM study was a longitudinal qualitative cohort study conducted in Gugulethu, Cape Town, South Africa. Gugulethu is a former Black South African township with a high prevalence rate of antenatal HIV infection (30%) and high levels of poverty [26].

### Recruitment and sample selection

Participants were recruited during pregnancy from the Gugulethu Midwife Obstetrics Unit (MOU), which forms part of the Gugulethu Community Health Centre, a public sector facility. The MOU serves nearly 5000 pregnant and postpartum women annually. Eligibility for the study included being: (1) 18 years or older, (2) pregnant based on clinic records and estimated to be in her 8th month of pregnancy (32–35 weeks pregnant), (3) HIV positive based on clinic records and currently prescribed ART and (4) isiXhosa or English speaking. Women were excluded from the study if: (1) their pregnancy was categorized as high-risk for reasons other than HIV status (e.g. pre-eclampsia, preterm labor), (2) self-reported participation in another ART adherence-related study, or (3) unable to understand the consent process. Women were recruited from the clinic using structured purposive heterogenous sampling to ensure maximum diversity on age (18–24; >24 years), parity (1st pregnancy; previously pregnant), and education (non-matriculated; matriculated) [27, 28].

### Longitudinal qualitative cohort procedures

Enrolled participants completed 4 interviews spanning late pregnancy through up to 12 months postpartum. Each interview lasted 1 to 1.5 h and was conducted in either isiXhosa or English depending on preference and was recorded using a digital voice recorder. All interviews were conducted by a trained and experienced qualitative research assistant for whom isiXhosa is her native language and who is also fluent in English. The interviewer identifed as a Black Xhosa woman and resided in the community. All interviews took place in a private research office separate from but in close proximity to the MOU. Interviews were then transcribed and if necessary, translated into English by a second first language research assistant. Translated portions were checked by bilingual research staff to ensure proper translation and meaning. Weekly supervision meetings were conducted with the principle investigator and the research assistant to debrief each interview, discuss emergent results, and to guide the iterative nature of the interviews.

#### Timepoints and content

Participants were first interviewed during their eighth month of pregnancy and then were followed through childbirth and interviewed again when their children were 6 to 8 weeks, 4 to 6 months, and 9 to 12 months old, capturing key points of transition that may influence HIV care decisions and behaviors. Interviews for each time point followed a semi-structured guide, which probed for the key components of Transition Theory that may impact ART adherence and engagement in HIV care.

During the last interview (9–12 months postpartum), participants were also asked about their preferences for potential ART adherence behavioral intervention components, formats, and content and for their reasoning behind those preferences. We specifically inquired about participant preferences for groups versus individual interventions, length of intervention (e.g. number of sessions, length of individual sessions), and time frame (e.g. when during pregnancy and/or postpartum). Following these questions, we also provided them with a list of potential topics and asked, “*what are the top 3 most important things you think a program for postpartum women living with HIV should cover?*” They were then asked to explain their choices. The potential topics came from a rapid thematic analysis of the first three sets of interviews with this cohort and included (a) education about HIV, PMTCT, and how medications (ART) work in the body, (b) HIV care transfer process, (c) navigating relationships with partners/how to have healthy relationships, (d) HIV status disclosure to partners/family/friends, (e) employment, (f) baby – infant care, breastfeeding, nevirapine, infant testing, (g) family planning/ contraception, (h) mental health/depression, (i) violence/intimate partner violence, (j) medication adherence behavioral strategies, k) discussing stigma, l) substance use, and m) social support. The order of the topics was presented randomly.

#### Ethical approval and informed consent

This study was approved by the University of Cape Town Faculty of Health Sciences Human Research Ethics Committee (HREC Ref #: 344/2017) and Brown University Institutional Review Board through an IRB Authorization Agreement (IAA #17–45). Informed consent was completed with all women enrolled in the study. All study procedures comply with international research standards as set forth by the Declaration of Helsinki.

### Qualitative data analysis for intervention development

Transcripts were analyzed in NVivo 12. We used an inductive thematic coding approach in conjunction with ‘sensitizing concepts’ to guide our analysis [29]. Specific analysis techniques included open coding, axial coding, marginal remarks, comparisons, and memo-writing. Sensitizing concepts allow researchers to start with a general reference point to guide interpretation of empirical data while maintaining the use of inductive analysis to allow themes to emerge from the coded data. The use of sensitizing concepts in conjunction with an inductive approach allowed us to use Transition Theory as a starting off point for data analysis and interpretation, while also allowing for emergent codes to arise from the data that did not fit directly into Transition Theory constructs to create a comprehensive picture of women’s lived experiences. JP and DJ read through the transcripts and created an initial codebook based on the transcripts and Transition Theory. During coding if an emergent code was identified, this was discussed among the coding team and previously coded transcripts were recoded to ensure any new concepts were adequately captured. Themes were then generated from these codes to describe women’s experiences of transition from pregnancy to postpartum and the impact of ART adherence and engagement in care. Additionally, for the Time 4 data on intervention design and content (the focus of this analysis), we quantified some responses using counts to further comprehend participant preferences and inform intervention development.

## Results of the PAM study

We briefly describe the specific findings of the PAM study that informed the intervention content and focus on new data from Time 4 regarding participant preferences for intervention design. Table 1 provides demographic information for the participants enrolled in the longitudinal qualitative cohort.

**Table 1:** Demographic Characteristics of Participants in the PAM Study at Enrollment (8th month of pregnancy)

Demographics	N (%)
Black	30 (100%)
Cultural Identity	
Xhosa	29 (96%)
Shona	1 (3%)
Primigravida	10 (33%)
Completed secondary education	9 (30%)
Household Income (per month)	
Less than R1000 ($70)	9 (30%)
R1000-R5000 ($70–350)	17 (57%)
R5000 or greater	4 (13%)
Average Self-Reported ART Adherence (Visual Analog Scale)	M = 92.86% (SD = 10.87)

### Summary of PAM study qualitative findings

Analyses of the data from the longitudinal qualitative cohort reveal a variety of facilitators and barriers to ART adherence during the transition from pregnancy to postpartum. Preliminary results from the pregnancy interview showed high adherence motivation. Anticipated barriers postpartum were employment/financial concerns, logistical concerns around childcare and breastfeeding, worries about vertical transmission and difficulties bonding. Additional concerns included forgetting their ART medications and confusion about where to receive HIV care postpartum. At six weeks postpartum, women reported experiencing many of the barriers that they had anticipated, particularly financial and logistical challenges. ART adherence motivation remained high postpartum, but some women reported issues remembering to take medications while caring for infants. Factors that appeared to facilitate the transition from pregnancy to postpartum with respect to ART adherence include supportive partners and families during pregnancy and postpartum and a sense of preparation during pregnancy. Factors that seemed to inhibit HIV engagement in care and ART adherence include unsupportive partners or a change in partner support from pregnancy to postpartum and lack of financial support. Many of these factors directly map onto key conditions outlined by Transition Theory including socioeconomic status, preparing for the infants’ arrival, and community factors including family and partner support.

### Intervention content findings

Of the 30 participants enrolled in the study, 25 (86%) completed an interview at Time 4 (9–12 months postpartum). Participants were presented with 13 key topics that had been identified in the prior three interviews as common issues that potentially impact HIV treatment adherence amongst this group of women. These key topics were presented in a random order and participants were asked to choose the top three topics they felt that an intervention should address (Table 2). If the participant was confused about a particular topic, the interviewer provided a more detailed explanation and examples of the potential content. Education about HIV, PMTCT, and ART medications were selected as the top content areas by a majority of women (N = 15/25;60%). One participant stated:

*The HIV topic is important as well, because we go through different physical experiences, it is important to know what other people do to ensure that they adhere to their treatment and educate those around them. (PID 121, 34 years old, multiparous).*

Baby care, including breastfeeding, nevirapine, and HIV testing for infants were also selected as top content areas by many women (N = 12/25; 48%). When choosing this topic, one participant highlighted challenges associated with breastfeeding that illuminate a need for support:

*As for baby –infant care and breastfeeding; it is important to breastfeed although many mothers do not want to breastfeed their babies. They breastfeed only when they are still admitted and stop it after they have been discharged and their babies get infected with HIV because they feed them with food which is not good for their health. Some of them live in dirty communities like me so it is not healthy to bottle feed their babies because flies may also spread germs over their baby bottles. Breastfeeding is safer than formula feeding.* (PID 122, 25 years old, multiparous).

The next most common area was violence/intimate partner violence (IPV; N = 7/25; 28%). One participant noted how prevalent IPV is in her community: *“Intimate partner violence is very common and to some people it happens while they are pregnant so they should be advised on how to deal with such situations.”* (PID 110). The next two most common areas were mental health/depression (N = 6/25; 24%) and employment (N = 6/25; 24%). One participant discussed how these two issues could also be related:

*Mental health and depression… the thing is you don’t know when you suffer from stress or depression. You think worrying too much is normal, but it kills you. I also worry about working the whole month and getting paid less.* (PID 126, 24 years old; primiparous).

Family planning/contraception, medication adherence, and social support were chosen by 20% of the participants. The topics selected less often were the HIV care transfer process (N = 1/25; 4%), healthy partner relationships (N = 2/25; 8%), disclosure (N = 3/25; 12%), discussing stigma (N = 2/25; 8%) and substance use (N = 4/25; 16%).

**Table 2:** Counts of Qualitative Responses to Content and Design Preferences

*Potential Topics for Hypothetical Program*	N	%*
Education about HIV, PMTCT, and medications (ART)	15	60
Baby – infant care, breastfeeding, nevirapine, infant testing	12	48
Violence/intimate partner violence	7	28
Employment	6	24
Mental health/depression	6	24
Family planning/ contraception	5	20
Social support	5	20
Medication Adherence (strategies for taking medications on time)	5	20
Substance use	4	16
Disclosure to partners/family/friends	3	12
Discussing stigma	2	8
Navigating relationships with partners/how to have healthy relationships	2	8
HIV care transfer process	1	4
***Preference for Individual vs. Group Intervention***		
Group	21	84
Individual	4	16

*Note: Participants were encouraged to indicate their top 3 most important issues a program should cover

### Intervention format

Participants were asked about their preferences with regards to the format of the intervention.

#### Groups vs. individual sessions

Most participants preferred that the intervention be delivered to a group of women rather than individually (N = 21; 84%). Many participants said that they would like to hear about the experiences of other women like them:

*I would choose the one that includes all mothers because I would hear different views and challenges. Maybe we might talk about experiences that we couldn’t handle in our lives, but I will learn how to tackle them in the future. I would get to learn more about their different challenges. I would enjoy being in a group.* (PID 130, 28 years old; primiparous)

Other participants noted that they didn’t know many other women living with HIV and groups would allow them to meet other women like them in their community. One participant said:

*I would prefer a program with a small group of 4–8 women living with HIV but I don’t know of any other women who are living with HIV in my community. I think I know only one woman who is living with HIV in my community since I met her in (the local) clinic when I went there for my HIV care.* (PID 111, 24 years old; multiparous).

Conversely, there were some women that preferred an individual based intervention (N = 4; 16%). Reasons for this preference included a need for privacy and worries that speaking openly in a group could be difficult. This is evidenced by participant 101 who initially thought she would prefer a group format but as she talked through this option with the interviewer realized she would personally prefer a one-on-one format.

*I prefer to join a small group to get experience on how other mothers manage to cope with situations they come across. I would go for something which will be short term. I think I have a fear about being reminded about my past mistakes. I don’t mind to listen to other people’s horrors but I am not comfortable to share mine. I don’t want to be a part and parcel of support group unless I go there anonymously just to listen to what other mothers are going to say. I think a one-on-one program could suit me better.* (PID 101, 40 years old; multiparous)

#### Length of time

In terms of women’s preference for a particular number of sessions for the intervention, most women wanted to meet multiple times, rather than just once. Many pointed out that this would give women the opportunity to get comfortable with others in a group format as well as establish rapport when one-on-one with a counselor: *“I would like to meet them several times because they might avail themselves in different times for some reasons.”* (PID 104, 38 years old; multiparous).

The preferred length of time for each session varied with most women wanting one hour to 2–3 h. Some women wanted more time to give women a chance to settle into the meetings and talk to one another:

*Interviewer: Why do you choose to spend 2 h with them?*

*Participant 111: It is fine to accommodate mothers who want to share their ideas and opinions. We must give a chance and to listen to each other without a hurry.*

Other women wanted a shorter meeting time that could fit into their other many commitments.

*One hour would be enough.…We have other things to do such as cooking for our families and to take care of our babies; you can leave your babies with other people for more than 2–3 h more especially if you are not going to pay them. (P116, 34 years old;* multiparous*)*

#### When should the intervention occur?

Participants were also specifically asked whether intervention sessions should occur during pregnancy, postpartum, or both. Many women underscored the importance of gaining information and support in pregnancy:

*I think these group meetings would be convenient while people are pregnant, some people get to know their status while they are pregnant. Understanding that people do not handle situations in the same way. Some people would want to harm themselves when they are informed of their HIV status, so these group meetings will help guide and empower HIV positive mothers and educate them at the same time about what to expect during their pregnancy. For me it’s better to have such interactions during pregnancy. (PID 111, 24 years old;* multiparous*)*

Women also recognized that continuing sessions that start in pregnancy through the postpartum would be helpful to address new concerns that arise, such as taking care of a newborn.

*I don’t know but I think when they are pregnant because everything such as HIV testing starts when a woman is pregnant. Mothers should start taking their HIV medications when they are pregnant and during postpartum; in fact they should take their medications for the rest of their lives. I once came across a pregnant woman who told me that she will stop taking HIV medications during postpartum. People like her need to be motivated to take their HIV medications because she is ignorant about being HIV positive. (PID 119, 26 years old;* multiparous*)*

Taken together, findings from these qualitative analyses were used to inform the format and content of the behavioral intervention.

### *Womandla Health* intervention

**Fig. 1:**
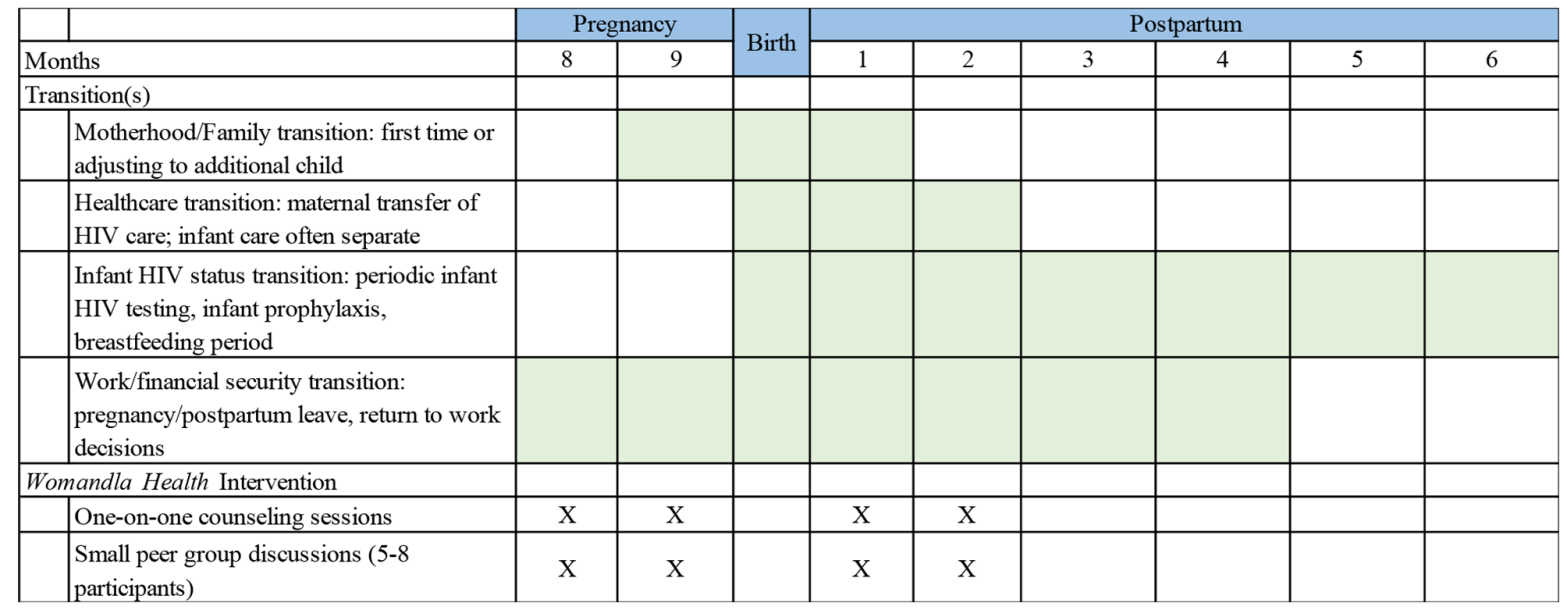
Pregnancy/Postpartum Transitions and *Womandla Health* Biobehavioral Intervention Sessions

The intervention development process was guided by Fernandez et al.’s Intervention Mapping process [30]. Informed by the qualitative data, the wider literature on ART adherence, and Transition Theory, we developed a logic model of the problem (Step 1) and logic model of change (Step 2) to identify key determinants of change to improve ART adherence and retention in HIV care in the postpartum period. Following these steps, we engaged in Step 3 – Program Design – which focuses on the generation of program themes and finalizing intervention components, scope and sequence of intervention activities [30]. Utilizing the information provided by participants in the longitudinal qualitative cohort, the research team designed a multicomponent, lay health worker delivered bio-behavioral intervention, called *Womandla Health* (Fig. 1). The word “womandla” is a variation on the isiXhosa word “amandla” (power and strength) which evokes a sense of women’s empowerment among Black South Africans. The *Womandla Health* intervention seeks to support these same ideas of women’s strength and power in leading healthy lives for themselves and their children.

Briefly, this intervention spans late pregnancy and early postpartum and consists of monthly one-on-one sessions with a lay health worker for 4 months as well as monthly small group sessions with other participants for 4 months; more detail on the content of these sessions appears below. The intervention spans both pregnancy and postpartum because participants suggested that both time frames are important for providing support for women. Although the majority of participants preferred the group structure in order to foster peer support, the study team felt that it was necessary to also include an individual component to address the needs of those participants who may be uncomfortable sharing about certain topics (such as IPV) in a group setting. These sessions are in addition to regular HIV and antenatal care that women received through routine care services. The design and content of the intervention conditions are informed by Transition Theory and the qualitative data from the PAM study which highlight that the transitions conditions most salient for this population are socioeconomic status, preparing for the infants’ arrival, and community factors including family and partner support.

#### Lay health worker model

The lay health worker conducts all the one-on-one sessions as well as assisting in facilitating the peer group sessions. The intial test of the intervention utilizes a lay health worker who was trained as a community health worker in the South African health care system but is hired independently by the research team to serve as a test of concept prior to planned integration into the health system. Our comprehensive review of the literature [21] indicates that interventions that included a minor alteration of the healthcare system were also more effective than interventions that did not; examples of types of alterations of the healthcare system included in this review were task-shifting from physician centered care to nurse/peer counselor care as well as the use of trained lay workers to deliver education, monitor barriers to ART adherence and to provide assistance in accessing healthcare, including accompanying women to the district hospital. Another recent review of HIV specific maternal and child health programs delivered by lay health workers in Africa found improvements on community awareness of mother-to-child transmission, condom use, clinic attendance/retention in care, and infant testing [31]. Current South African community health worker education does not include integrated training in maternal and child health and ART adherence focused on the transition from pregnancy to postpartum. This intervention provides additional training to lay health workers in these content areas.

#### Multicomponent intervention

Given the wide ranging and multi-level factors that both inhibit and facilitate successful transitions from pregnancy to postpartum and subsequent adherence to ART, we recognized the need for a flexible intervention that could address the varying issues arising in women’s lives. The multicomponent design of this study allows for several topics common to many women to be discussed within the group setting but also allows for individual tailoring in the one-on-one session with the lay health worker, particularly for topics that may be sensitive to discuss in a group setting, such as substance use or IPV. Given the mixed findings from the qualitative work with women expressing concerns about accessibility and privacy, an mhealth component was not included in the design of this intervention.

**Individual Component.** Our intervention design includes a one-on-one component with a lay health worker who, using motivational interviewing techniques, helps the participant identify her own individual needs and her self-identified strategies for overcoming any barriers she is facing. These barriers could be directly related to ART and HIV care or could be more indirectly related to these issues, such financial issues or childcare. Utilizing motivational interviewing, in addition to a manual, allows for a more flexible approach and for counseling sessions to be more engaging and guided by patients’ own strengths, limitations, and motivations [32–34]. Motivational interviewing has been shown to be improve health outcomes in the South African context, among pregnant/postpartum and among individuals living with HIV [35–39]. The lay health worker is trained in motivational interviewing as part of the training for the *Womandla Health* intervention. Key elements of motivational interviewing that were focused on in this training included asking open ended questions, providing affirmation of participants strengths and past successes, empathetic listening, providing reflections and summaries of what participants say to establish shared understanding [40]. The lay health worker completed two weeks of intensive training that included 10 h of role playing and feedback on performance. During the study, the lay health worker received weekly supervision and “spot checks” of counseling session audio recordings to monitor and reinforce adherence to key elements of motivational interveiwing.

Each individual session lasts approximately one hour to address the need of women to fit the intervention into their busy schedules, as noted by participants in the qualitative interviews. Each of the four sessions begin with guiding prompts and topics to orient the session but the structure of the sessions is intentionally flexible, such that prompts around these topical areas can lead to broader discussions of the topics and issues that are the most salient for individual women. Session 1 focuses on motherhood and preparation for the new baby, session 2 focuses on discussions of ART adherence and engagement in HIV care, and disclosure, session 3 focuses on self-care postpartum and discussions of labor experiences, and session 4 focuses on existing support systems and how to leverage partners and families to support ART adherence and HIV care and living “positively”. At the end of each session, the lay health worker also assists the participant in completing a goal setting worksheet, which focuses on translating the discussions of the session to actionable goals and priority setting. In addition to the four sessions, the community health worker are also able to provide tangible support both in terms of referrals to other services (social services, governmental aid programs) as well as physically assisting with linkage to care and navigating clinical environments depending upon the needs of each individual participant.

**Group Component.** Most women in the qualitative study preferred a group component of the intervention. Our comprehensive review of the literature [21] also indicates that interventions that have a group component are more effective for improving ART adherence. Topical areas mirror some of the topics covered in the individual component to introduce these topics in the individual sessions to prepare women for group discussion and to allow for reflection on group discussions in the one-on-one sessions. Drawing on the qualitative findings of common barriers for women as well as important transition conditions posited by Transitions Theory, session 1 focuses on brief education about HIV and PMCTC and discussion of motherhood, session 2 focuses on preparation for baby, session 3 focuses on life with baby (balancing self-care and baby care), and session 4 focuses on dreaming about and planning for the future (education, employment, and family planning). Similar to the individual component, the session guides start with guiding prompts and topics outlined above but are flexible to allow for broader discussions about issues that are salient to women in the group. These groups last 2-2.5 h, are facilitated by the lay health worker and are relatively small with 5–8 women per group. The length of the sessions was determined through the qualitative data in which some women mentioned the utility of 2–3 h sessions so that there would be time to settle in and get comfortable with other people in the group.

## Discussion

The development and evaluation of novel interventions for women living with HIV during the postpartum period remains vital to achieving the full benefits of Option B + programs. The design of the *Womandla Health* intervention is grounded in the expressed needs of postpartum women as well as the existing behavioral intervention literature for women living with HIV [21]. Utilizing Transition Theory as a foundation, the *Womandla Health* intervention focuses on the critical transition points that occur during late pregnancy and early postpartum while also considering the unique social context of this period in which partners, families, and communities play large roles in the well-being of mothers.

The *Womandla Health* intervention is one of the first biobehavioral interventions designed specifically for women living with HIV as they transition from pregnancy to postpartum to address ART adherence and engagement in care and is the only intervention which utilizes Transitions Theory as its theoretical framework [21]. Previous interventions for this population have focused on technology-based or mHealth approaches (such as SMS reminders or telehealth counseling), which given our mixed qualitative results were not a good fit for our population [41, 42]. Furthermore, previous ART interventions for pregnant and postpartum women have not used group components, which have been shown to have larger effects on ART adherence among general populations of women living with HIV [21]. Bringing together these important considerations will hopefully lead to impactful public health results among our population.

The *Womandla Health* intervention will be initially tested using a 1:1 small-scale randomized controlled trial design. The intervention will be compared to an enhanced standard of care group which will receive one session with a lay health worker to provide basic education and to simulate currently available services by CHWs. Self-reported ART adherence, retention in HIV care services, and viral suppression based on clinic records will be assessed at 6 months postpartum. Following the RCT, women randomized to the *Womandla Health* intervention will be interviewed to assess participants’ experiences of the intervention, perceived utility, and identify ways to refine the intervention for future evaluation. The Womandla Health intervention will be considered successful if there is a moderate effect of the intervention on self-reported ART adherence compared to the control. Given the small sample size and timing of follow-up, it may be difficult to see significant effects of the intervention on the objective outcomes of retention in care and viral suppression. If successful, findings will provide an innovative approach to HIV treatment by capitalizing on the transition into motherhood to bolster self-care behaviors, focusing on ART adherence and also women’s overall postpartum health and psychosocial needs.

## Data Availability

The dataset used is available from the corresponding author on reasonable request.
